# Comment on ‘Prevalence of depression in patients with sarcopenia and correlation between the two diseases: systematic review and meta‐analysis’

**DOI:** 10.1002/jcsm.12983

**Published:** 2022-03-17

**Authors:** Fan Zhang, Hui Wang, Huachun Zhang

**Affiliations:** ^1^ Department of Nephrology Longhua Hospital Shanghai University of Traditional Chinese Medicine Shanghai China; ^2^ Department of Anorectology Longhua Hospital Shanghai University of Traditional Chinese Medicine Shanghai China; ^3^ Department of Nursing Longhua Hospital Shanghai University of Traditional Chinese Medicine Shanghai China

We read with interest the recent publication by Li *et al*.,^1^ which aimed to investigate the prevalence of depression among individuals with sarcopenia and determine whether sarcopenia was independently associated with depression. The results showed that the overall prevalence of depression was 0.28 [95% confidence interval (*CI*): 0.21–0.36], and the overall adjusted odds ratio (OR) between sarcopenia and depression was 1.57 (95% *CI*: 1.32–1.86). However, we would like to state the following methodological issues.

First, we thought that the data extracted by the authors might be incorrect: (1) the authors extracted a sample size of 50 individuals with sarcopenia and 32 patients with depression, respectively, from Olgun Yazar and Yazar^2^; however, in fact, 116 individuals with sarcopenia were reported by Olgun Yazar and Yazar; (2) the prevalence of depression among patients with sarcopenia from Hsu *et al*.^3^ was 28 cases, but the authors included 32 cases for analysis. According to the authors' data, the overall sample size decreased relative to the actual reported number, while the number of depressions increased, and the results of their meta‐analysis of prevalence became higher.

Second, the forest plot in *Figure* 2 of the original article was generated by the authors based on the *metan* command of the Stata software, but in fact, the results of the meta‐analysis of single group rates using the *metaprop* command may be more realistic; the latter was published by Nyaga *et al*.^4^ in 2014 to combine the rates of multiple studies in a meta‐analysis. Based on this, we replotted the forest plot using ‘metaprop case n,random second (fixed) cimethod (exact) lcols (author year) xlab(0.2,0.4,0.6,0.8) dp(3)’, and the results showed that the *Q* statistic = 134.910, corresponding *P* < 0.001; *I* ^2^ = 89.623%, suggesting heterogeneity among studies and the prevalence of depression based on a random‐effects model was 25.6% (95% *CI*: 19.3–31.8%) (*Figure* 1), slightly lower than the results reported by Li *et al*.^1^

**Figure 1:**
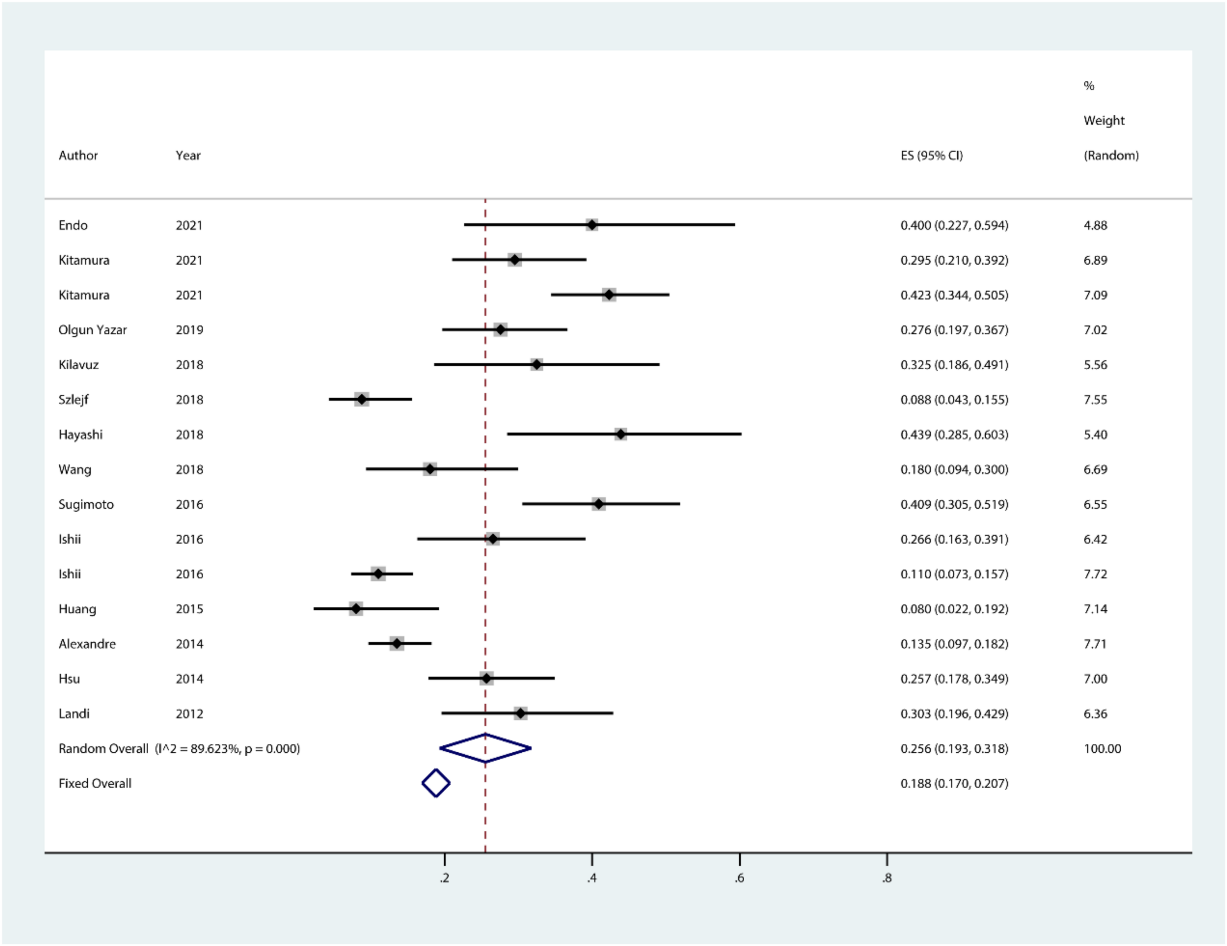
Forest plot of prevalence of depression in sarcopenia. CI, confidence interval; ES, effect size.

Third, we note that the study published by Kitamura *et al*.^5^ reported the overall population's multiple logistic regression analysis results [OR: 1.9 (95% *CI*: 1.4–2.6)], but the authors extracted data for men and women separately for inclusion in the analysis; the results of our re‐perform meta‐analysis showed that the overall adjusted OR between sarcopenia and depression was 1.565 (95% *CI*: 1.315–1.862) (Supporting Information, *Figure* S1), with considerable heterogeneity (*P*_heterogeneity_ < 0.001; *I* ^2^ = 75.7%). Although this result is comparable with the authors' effect size, the results of the analysis incorporating data from the total population reported by the original authors may be more relevant and accurate.

In addition to the methodological issues mentioned above, the heterogeneity of the meta‐analyses reported in the original article was considerable. We thought that the authors should conduct further meta‐regression to determine the sources of heterogeneity among studies. In addition, assessment of publication bias by graphical method (funnel plot) or quantitative analysis (Begg's and Egger's test) is also an indispensable part of a meta‐analysis.

Despite the methodological shortcomings, we acknowledge that the results of the study conducted by the authors should draw the attention of medical professionals to the fact that depression is present in approximately one‐third of individuals with sarcopenia. In other words, it is essential not to neglect psychological issues such as depression while addressing muscle dysfunction.

## Supporting information




**Figure S1.** Forest plot of the adjusted ORs between sarcopenia and depression.
