# Supplementary figures and images for: Comment on ‘Prevalence of depression in patients with sarcopenia and correlation between the two diseases: systematic review and meta‐analysis’
Source: J Cachexia Sarcopenia Muscle. 2022 Mar 17;13(3):1956–7. doi: 10.1002/jcsm.12983 (PMC9178152; doi:10.1002/jcsm.12983)

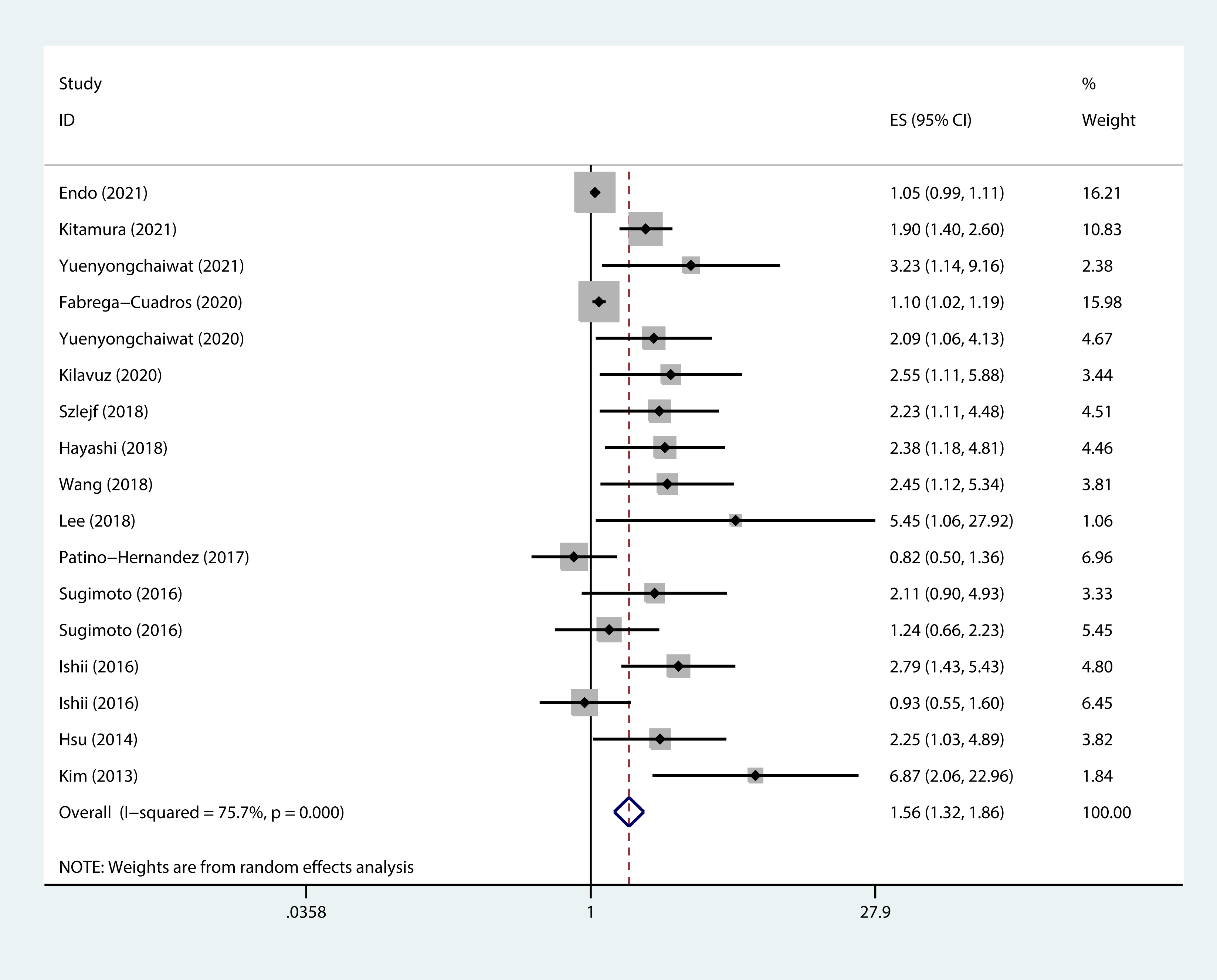

Supplement: Supplementary file 1 — Figure S1. Forest plot of the adjusted ORs between sarcopenia and depression. [file JCSM-13-1956-s001.tif]
